# CRISPR/Cas9-Directed Gene Trap Constitutes a Selection System for Corrected *BCR/ABL* Leukemic Cells in CML

**DOI:** 10.3390/ijms23126386

**Published:** 2022-06-07

**Authors:** Elena Vuelta, José L. Ordoñez, David J. Sanz, Sandra Ballesteros, Jesús M. Hernández-Rivas, Lucía Méndez-Sánchez, Manuel Sánchez-Martín, Ignacio García-Tuñón

**Affiliations:** 1 Departamento de Medicina, Universidad de Salamanca, 37007 Salamanca, Spain; elena.vuelta.r@usal.es (E.V.); sballesteros@usal.es (S.B.); jmhr@usal.es (J.M.H.-R.); 2 Unidad de Diagnóstico Molecular y Celular del Cáncer, Instituto Biología Molecular y Celular del Cáncer (USAL/CSIC), 37007 Salamanca, Spain; davidjsanz@gmail.com; 3 Servicio de Transgénesis, NUCLEUS, Universidad de Salamanca, 37007 Salamanca, Spain; mendez_lucia@usal.es; 4 Instituto de Investigación Biomédica de Salamanca (IBSAL), 37007 Salamanca, Spain; 5 Departamento de Fisiología y Farmacología, Facultad de Farmacia, Universidad de Salamanca, 37007 Salamanca, Spain; jlog@usal.es; 6 Servicio de Hematología, Hospital Universitario de Salamanca, 37007 Salamanca, Spain

**Keywords:** chronic myeloid leukaemia, BCR/ABL, CRISPR, gene therapy, CRISPR-Trap

## Abstract

Chronic myeloid leukaemia (CML) is a haematological neoplasm driven by the *BCR/ABL* fusion oncogene. The monogenic aspect of the disease and the feasibility of ex vivo therapies in haematological disorders make CML an excellent candidate for gene therapy strategies. The ability to abolish any coding sequence by CRISPR-Cas9 nucleases offers a powerful therapeutic opportunity to CML patients. However, a definitive cure can only be achieved when only CRISPR-edited cells are selected. A gene-trapping approach combined with CRISPR technology would be an ideal approach to ensure this. Here, we developed a CRISPR-Trap strategy that efficiently inserts a donor gene trap (SA-CMV-Venus) cassette into the *BCR/ABL*-specific fusion point in the CML K562 human cell line. The trapping cassette interrupts the oncogene coding sequence and expresses a reporter gene that enables the selection of edited cells. Quantitative mRNA expression analyses showed significantly higher level of expression of the *BCR/Venus* allele coupled with a drastically lower level of *BCR/ABL* expression in *Venus*+ cell fractions. Functional in vitro experiments showed cell proliferation arrest and apoptosis in selected *Venus*+ cells. Finally, xenograft experiments with the selected *Venus*+ cells showed a large reduction in tumour growth, thereby demonstrating a therapeutic benefit in vivo. This study represents proof of concept for the therapeutic potential of a CRISPR-Trap system as a novel strategy for gene elimination in haematological neoplasms.

## 1. Introduction

Chronic myeloid leukaemia (CML) is one of the best-known haematological malignancies. Originating in the stem cell compartment, the cytogenetic hallmark of the disease is the translocation between the t(9;22)(q34;q11.2), which results in the formation of the abnormal Philadelphia chromosome that harbours the *BCR/ABL* fusion oncogene [1,2]. Lifelong treatment with tyrosine kinase inhibitors (TKIs) remains the first-line therapy for the disease due to its effectiveness and the high remission rates it provides [3,4,5,6]. Unfortunately, the appearance of point mutations, leading to the development of TKI resistance during treatment, means that up to 33% of patients do not achieve an optimal response to treatment [5].

In the context of searching for new therapies, haematopoietic gene therapy has undergone unprecedented progress in terms of safety and efficacy [7,8,9,10]. The unique self-renewing and multi-potent properties of haematopoietic stem cells (HSCs), which can generate the entire haematopoietic lineage, make them ideal targets for gene correction of haematopoietic diseases. In addition, our considerable knowledge and experience about bone marrow autologous transplantation, which makes it possible to collect, genetically manipulate ex vivo and reinfuse the edited HSCs, would allow the evaluation and selection of correctly edited cells, improving the effectiveness of the process [9].

In this sense, CML is also an excellent candidate for evaluating gene therapy strategies, since it is well-established that all pathological features of the disease can be attributed to a single genetic event, the fusion of the *BCR* and *ABL* genes [11,12,13,14,15,16]. For this reason, many works have been published in recent years focusing on the genetic disruption of the *BCR/ABL* coding sequence at the genomic level using gene-editing tools, such as zinc finger [14] and, more recently, CRISPR/Cas9 nucleases [15,16,17], which provide the highest level of efficiency in HSC genomic editing [18]. These approaches are based on the ability of these nucleases to induce indel mutations at the target sequence, abrogating the expression of the fusion oncogene. However, the presence of unedited cells, due to the efficiency of CRISPR system and its delivery method, is responsible for a considerable limitation in its overall efficiency. Therefore, despite the promising results obtained, which demonstrate the therapeutic potential of the elimination of *BCR/ABL* in leukemic HSCs, even in bone marrow patient-derived xenografts [19], it is still necessary to develop a cell-editing selection method to turn this approach into a therapeutic reality.

As previously mentioned, one of the greatest challenges of ex vivo gene therapy in CML, as in other haematopoietic malignancies, is that posed by the existence of residual unedited cells with tumour activity that would promote post-transplantation relapse [20]. However, the nature of ex vivo gene therapy approaches could allow cell sorting of the edited HSCs prior to bone marrow transplantation (BMT).

The CRISPR-Trap approach was first described in 2018 by Reber S, et al., who designed a system for the generation of Knockout genes based on the introduction, through HDR, of a customizable cassette in the coding sequence of the gene, completely preventing expression of the ORF [21].

In this work, we evaluated the ability of a CRISPR-Trap system to direct by homology recombination (HDR) a gene targeting strategy for specifically trapping *BCR/ABL* oncogene expression. We show that this CRISPR-Trap assay abrogated oncogene expression by inserting a fluorescent reporter gene into the coding sequence of *BCR/ABL*, which would make possible to select solely the edited haematopoietic CML cells. The CRISPR-Trap system precisely selected the cells in which the oncogene had been properly disrupted. Importantly, xenograft assays with these sorted CRISPR-Trap-edited cells demonstrated a therapeutic benefit.

We demonstrate for the first time the feasibility of the CRISPR-Trap strategy for knocking out oncogenes and sorting edited cells, and thereby its value as a new tool in gene therapy approaches for treating haematological malignancies.

## 2. Results

### 2.1. The CRISPR/Cas9 System Efficiently Directs the Specific Integration of a Gene Trap Donor Cassette at the BCR/ABL Locus

**Figure 1:**
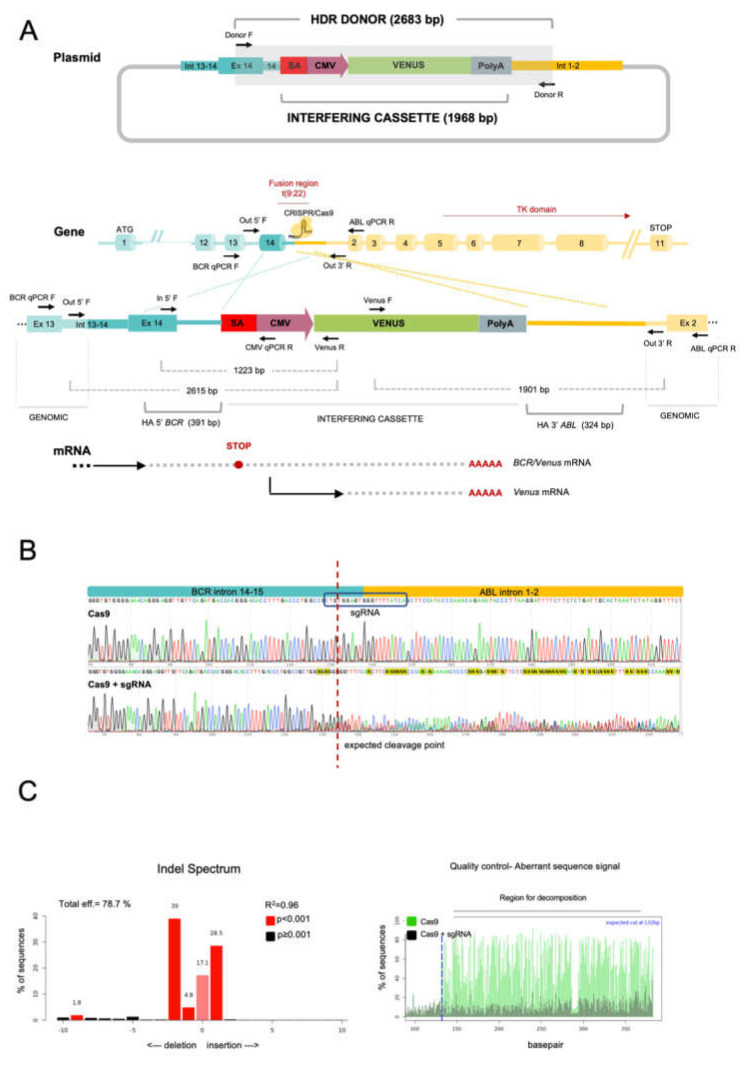
(**A**) Schematic representation of the CRISPR-Trap system plasmid and the target region of *BCR/ABL* fusion gene and the plasmid harboring the interfering cassette flanked by homology arms, containing a splicing acceptor sequence (SA), the CMV promoter (CMV) and the Venus fluorescent protein coding sequence. Black arrows represent oligos used to obtain the HDR donor molecule by PCR. CRISPR/Cas9 expected cut at BCR/ABL target sequence. The resulting *BCR/ABL* sequence after homology directed repair using interfering cassette as DNA donor is shown. Introduction of the SA-CMV-VENUS sequence into the intronic region of *BCR/ABL* disrupts the reading frame of the oncogene, also promoting expression of the Venus reporter. (**B**) Sanger sequencing of the CRISPR target sequence. Cells electroporated with CRISPR/Cas9 showed a mixture of sequences at the expected cleavage point (dotted red line). (**C**) TIDE decomposition algorithm analysis of the edited sequence in Cas9 + sgRNA cells, showing high editing efficiency at the expected cleavage point. The left panel illustrates the aberrant sequence signal in Cas9 control cells (black) and Cas9 + sgRNA-edited cells (green) and the expected cleavage site (vertical dotted line).

**Figure 2:**
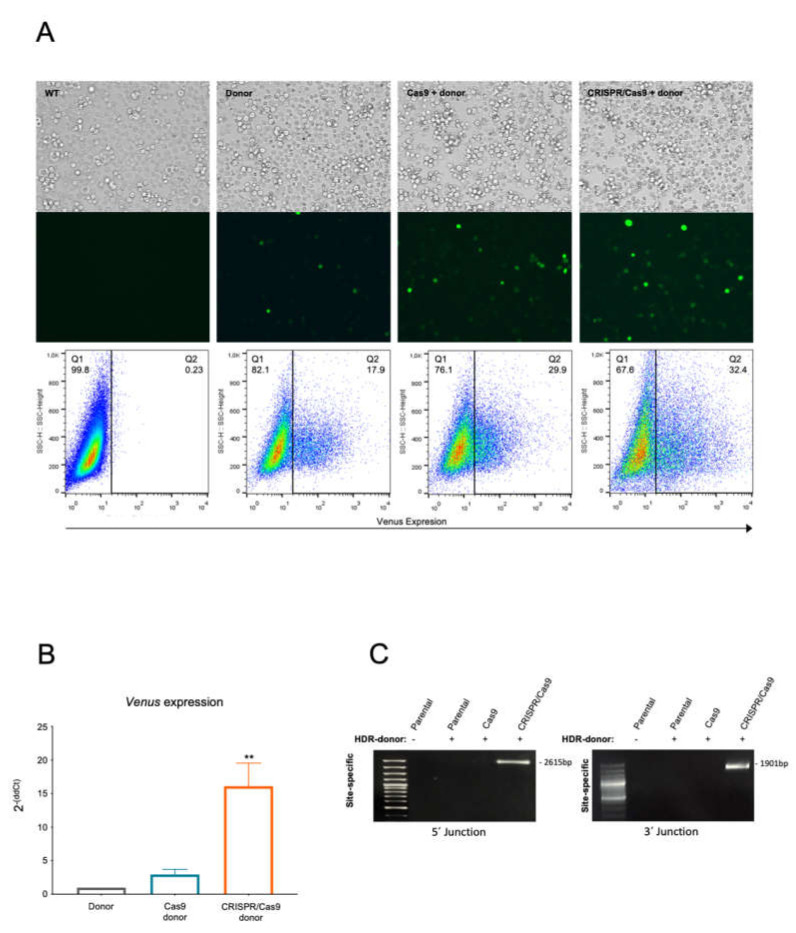
CRISPR-Trap system electroporation in K562 cells. (**A**) *Venus* expression K562 electroporated with HDR donor (used as control) and CRISPR-Trap system (CRISPR/Cas9-HDR donor), and Venus-positive cell quantification by flow cytometry of each condition. (**B**) *Venus* expression quantification by real-time PCR. (**C**) Molecular characterization of the CRISPR-Trap system. 5′ and 3′ junction site-specific PCR amplification in cells electroporated with CRISPR-Trap system in 5′(oligos: Out5′F/VenusR) and 3′ (oligos: VenusF/Out3′R junctions (2615 bp and 1901 bp, respectively). ** *p* < 0.01.

To explore the effects of a dsDNA HDR donor containing a high-expression CMV-Venus cassette (Figure 1A), the K562 cell line was divided into three experimental groups according to the conditions for their subsequent electroporation: (a) with the donor dsDNA (donor), (b) with the donor DNA and Cas9 nuclease without sgRNA (Cas9 + donor) and (c) with the donor DNA, Cas9 nuclease and the specific *BCR/ABL* sgRNA (CRISPR/Cas9 + donor). Twenty-four hours after electroporation, fluorescent cells (*Venus* + cells) were observed in all three groups, being most abundant in the CRISPR/Cas9 + donor group (32.4% vs. 23.9% in Cas9 + donor and 17.9% in donor) (Figure 2A).

Due to HDR dsDNA-induced fluorescence under all experimental conditions, we investigated possible differences in reporter expression levels by qPCR. We found the *Venus* mRNA levels in the CRISPR/Cas9 + donor group to be significantly higher in the CRISPR/Cas9 + donor group than in the donor and Cas9 + donor groups (Figure 2B).

The result of the 5′ arm (Out 5′F/Venus R, Table 1), 3′ arm (Venus F/Out 3′R, Table 1) and full-length (Out 5′F/Out3′R, Table 1) site-specific PCRs corroborated the correct insertion of the donor HDR dsDNA at the *BCR/ABL* target sequence only in the CRISPR/Cas9 + donor group, with no site-specific integration detected in any of the controls (Figure 2C; Supplementary Materials Figure S3). Furthermore, the subsequent Sanger sequencing of the PCR products confirmed the proper junction between the genomic DNA and the interference cassette (data not shown), corroborating the HDR-mediated insertion of the donor dsDNA into the CRISPR/Cas9 + donor group.

### 2.2. The BCR/ABL Trapped Allele Is Properly Expressed When the Expression of the Oncogenic Version Is Reduced

**Figure 3:**
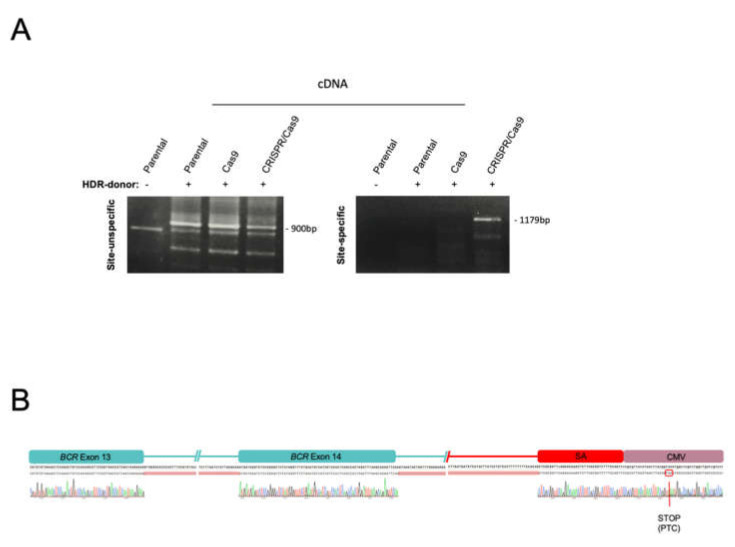
Expression analysis of target locus. (**A**) RT-PCR of *BCR/Venus* fusion RNA (900 bp site-unspecific, oligos: In5′F/VenusR; 1179 bp site-specific, oligos: BCRqPCRF/VenusR), in cells electroporated with the CRISPR-Trap system (CRISPR-Cas9 + HDR donor) and controls (Parental, Parental + donor, Cas9 + donor). (**B**) Sanger sequencing of site-specific RT-PCR corresponding to a *BCR/Venus* of K562 electroporated cells with the CRISPR-Trap system.

To verify the proper functionality of the CRISPR-Trap system, we studied all the possible mRNAs generated from the trapped allele. We designed a pair of oligonucleotides to amplify a region of the donor DNA (In 5′ F/Venus R; Table 1). This in-in RT-PCR showed a 900-bp band, corresponding to the size of the predicted *BCR/Venus* mRNA under all three experimental conditions (Figure 3A). Nevertheless, the site-specific in-out RT-PCR (BCR qPCR F/Venus R; Table 1) confirmed the correct expression of the cassette, specifically inserted into the *BCR/ABL* locus, only with the CRISPR/Cas9 + donor condition (Figure 3A). Interestingly, Sanger sequencing of the PCR products confirmed the existence of a processed BCR-CMV-Venus mRNA, containing a premature termination codon (PTC), which escapes the nonsense-mediated mRNA decay (NMD) signalling pathway (Figure 3B).

These results, coupled with the presence of fluorescent cells in the control groups, suggest an unspecific expression of the cassette when the dsDNA HDR donor was delivered either alone or with an “unguided” Cas9. As expected, site-specific HDR-mediated integration of the cassette was only detectable when all CRISPR/Cas9 trap reagents were delivered.

Having demonstrated the expression of the interfering cassette, we proceeded to quantify the expression levels of the *BCR/ABL* trapped locus versus native *BCR/ABL* allele in the pool of electroporated cells using a common forward oligonucleotide in the *BCR* sequence (BCR qPCR F) and two reverse oligonucleotides hybridizing in the ABL (ABL qPCR R) or CMV differential sequence (CMV qPCR R), respectively (Table 1).

**Figure 4:**
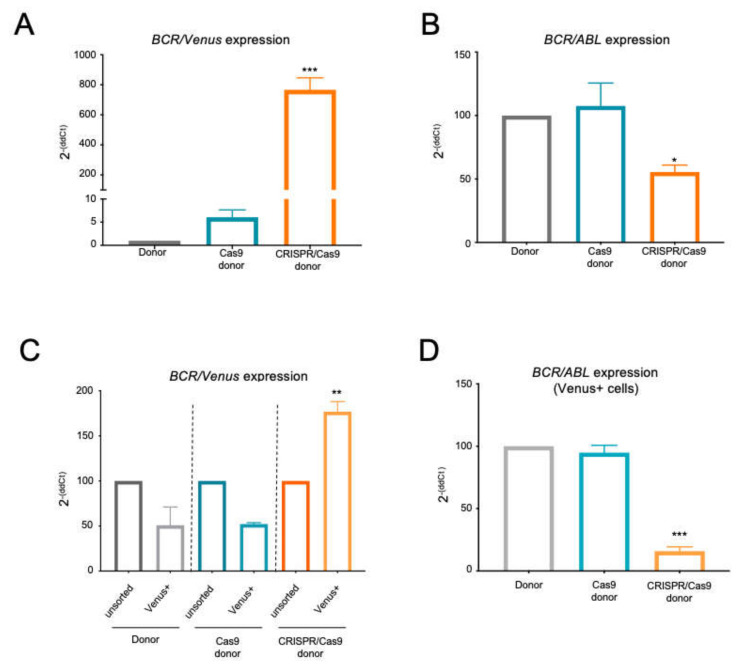
Expression analysis of targeted *BCR/ABL* locus. (**A**) qPCR of *BCR/Venus* and (**B**) BCR/ABL in K562 cells electroporated with the CRISPR-Trap system (CRISPR/Cas9 + donor) and controls (donor and Cas9 + Donor). (**C**) qPCR of *BCR/Venus* expression in electroporated K562 cells, comparing pool and *Venus*+ cells. (**D**) qPCR of *BCR/ABL* in Venus-positive cells electroporated with the CRISPR-Trap system (CRISPR/Cas9 + Donor) and controls (donor and Cas9 + donor) (mean ± SEM; *, *p* < 0.05; **, *p* < 0.01; ***, *p* < 0.001).

BCR/Venus qPCR confirmed the significant expression of the BCR-CMV-Venus allele solely in the CRISPR/Cas9 + donor group, in which it attained expression levels 800-fold greater than in the controls groups (Figure 4A). On the other hand, the BCR/ABL qPCR showed significantly lower oncogene mRNA levels in cells electroporated with the CRISPR-Trap system relative to control groups, thereby demonstrating the proper working of the gene trap when all the CRISPR/Cas9 components were present (Figure 4B).

### 2.3. The BCR/ABL CRISPR-Trap Enables the Selection of Gene-Targeted Cells

To examine whether the expression of the *Venus* reporter gene allows the successful selection of *BCR/ABL*-trapped cells, we compared the pool of electroporated cells in parallel with the sorted Venus fluorescent cell fraction from each experimental group. Selection of Venus fluorescent cells in the CRISPR-mediated group resulted in an almost two-fold higher level of expression of the *BCR/Venus* allele (176.9 ± 11.1% compared with the unsorted pool) (Figure 4C). However, no differences in *BCR/Venus* expression between pools and sorted cells were observed under any control conditions (Figure 4C).

Additionally, qPCR quantification of *BCR/ABL* mRNAs in Venus fluorescent cell fractions from all control groups showed high levels of oncogene expression (94.8 ± 5.9% of expression relative to the donor group), revealing the unspecific expression of the Venus cassette (Figure 4D). Conversely, Venus fluorescent cells sorted from the CRISPR/Cas9 + donor group showed significantly lower oncogene expression levels of up to 15.9 ± 3.5% relative to the control groups (Figure 4D).

### 2.4. The BCR/ABL CRISPR-Trap Promotes Apoptosis and Inhibits Proliferation in K562 Leukemic Cells

**Figure 5:**
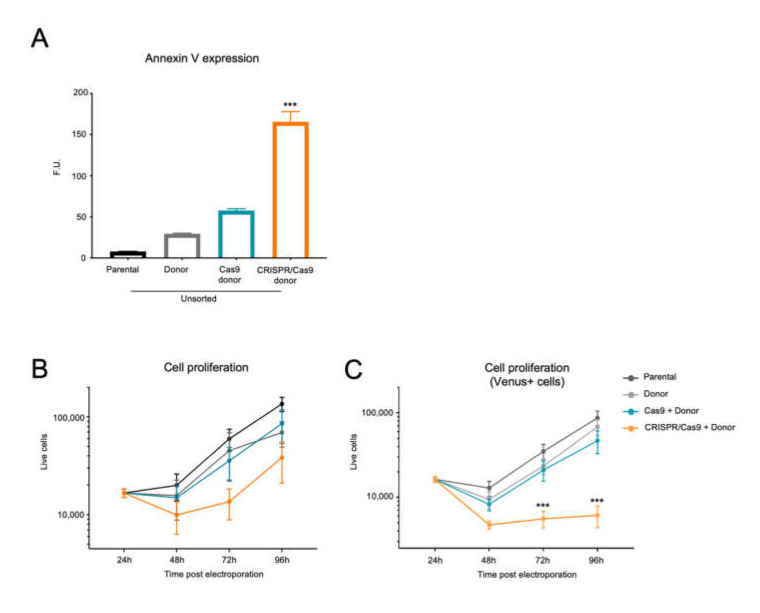
Functional analysis of CRISPR-Trapped *BCR/ABL*. (**A**) Annexin V expression analysis by flow cytometry of K562 cells 48 h after electroporation with the CRISPR-Trap system (CRISPR/Cas9 + Donor) and controls (Parental, Parental + Donor, Cas9 + donor). (**B**) Cell proliferation assay of K562 24 h after electroporation with CRISPR-Trap system and controls, and (**C**) Venus-positive sorted cells, over 96 h (mean ± SEM; ***, *p* < 0.001).

To evaluate the biological effect of the *BCR/ABL* CRISRP-Trap strategy, we measured the apoptotic levels and proliferative capacity of each group of electroporated cells. Forty-eight hours after electroporation, Annexin-V staining showed no significant differences between parental K562 cells (7.9 uf) and control groups (29.4 and 57.7 uf with the donor and Cas9 + donor conditions, respectively). In contrast, cells electroporated with the entire CRISPR-Trap system showed significantly higher levels of Annexin-V (165.4 uf) with respect to all control groups (Figure 5A).

The cell proliferation assay was carried out by seeding 1.5 × 10^4^ cells of each experimental condition 24 h after electroporation. At 48, 72 and 96 h, cells were counted using a cytometer. At 48 h, the control groups (donor and Cas9 + donor) had cell frequencies of 15,685 ± 6862 and 14,904 ± 4742, respectively, like that observed in K562 parental cells (19,946 ± 6035) (Figure 5B). However, cells from the CRISPR/Cas9 + donor group featured fewer cells (9936 ± 3602) at 48 h. After 72 h of culture similar raised frequencies of cells were observed for all control conditions (Figure 5B; parental: 59,896 ± 15,088; donor: 45,371 ± 23,313; Cas9 + donor: 35,689 ± 13,143). Nevertheless, this increase was lower in CRISPR-Trap electroporated cells (Figure 5B; 13,622 ± 4739). Finally, after 96 h of culture, a similar number of cells was observed for all conditions.

To investigate the tumour activity of CRISPR-Trap selected cells, a cell proliferation assay was performed using sorted parental cells and the Venus fluorescent cell fraction from all experimental conditions (Figure 5C). As expected, the Venus fluorescent cell fraction of both controls showed no differences in proliferation with respect to the sorted parental cells during 96 h of culture (parental: 59,896 ± 15,088; donor: 45,371 ± 23,313; Cas9 + donor: 35,689 ± 13,143). However, the Venus fluorescent cell fraction from the CRISPR/Cas9 + donor group revealed a substantially lower proliferation rate, with significantly fewer cells at 72 and 96 h than for the control groups (Figure 5C; 5572 ± 2465 and 6117 ± 3448, respectively).

### 2.5. The CRISPR-Trap System Prevents Tumour Activity of BCR/ABL, Thereby Producing a Therapeutic Effect in a CML Xenograft Model

**Figure 6:**
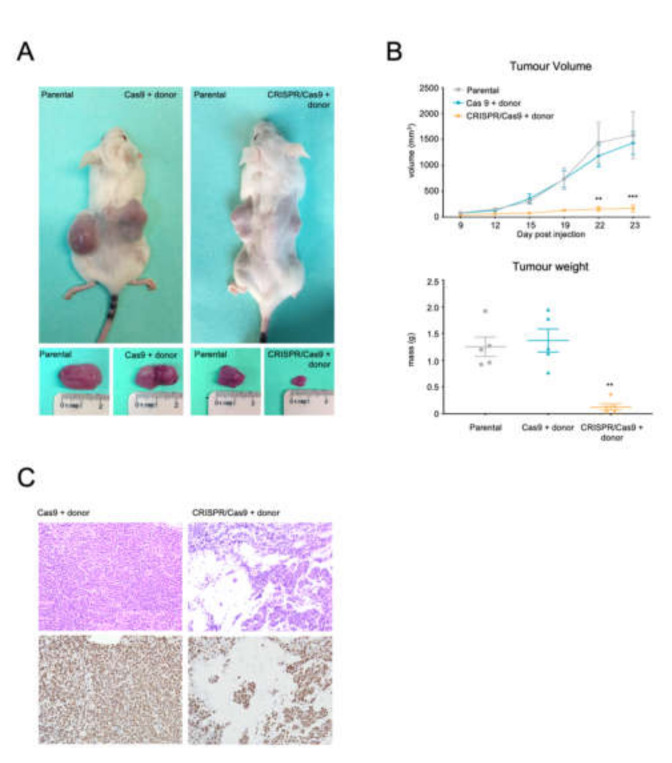
*In vivo* effects of CRISPR-trapped *BCR/ABL* oncogene. (**A**) External image of mice and developed tumors 23 days after subcutaneous cell injection. (**B**) Tumor growth (mm3) over the 23 days following subcutaneous cell injection. After 23 days, mice were sacrificed, and their tumor mass measured. The plots show means and SEM; ** *p* < 0.01, *** *p* < 0.001). (**C**) Histological analysis of tumors. Hematoxylin/eosin and Ki67 proliferation marker staining of tumor developed after Cas9 + donor and CRISPR/Cas9 + donor cell injection (20X magnification).

Finally, we studied the in vivo therapeutic effect of the CRISPR-Trap system in a CML xenograft model. 4 × 10^5^ Venus fluorescent cells from Cas9 + donor and CRISPR/Cas9 + donor groups, as well as 4 × 10^5^ parental cells, were sorted from the electroporated or parental pools, respectively. NSG mice were injected subcutaneously in both flanks with these cell suspensions, and tumours were allowed to develop for 23 days (Figure 6A).

Tumour growth over the 23 days post-cell injection revealed strong oncogenic activity in cells from the Cas9 + donor group, which gave rise to tumours similar to those of the parental K562 control group), reaching a volume of 1433.8 ± 219.6 and 1584.6 ± 453.8 mm^3^, respectively, after 23 days. Strikingly, the Venus fluorescent cells from the CRISPR/Cas9 + donor group developed tumours that were 90% smaller (166.8 ± 71.6 mm^3^ by 23 days post-injection) than those of the controls, implying that tumour activity was strongly inhibited in the cells selected by the CRISPR-Trap system (Figure 6B). Accordingly, after sacrifice at 23 days, the tumours had a significantly lower mass in the CRISPR-mediated condition than in the controls (0.13 ± 0.06 g vs. 1.3 ± 0.2 and 1.4 ± 0.2 g).

H&E staining also revealed significantly fewer tumour cells in *BCR/ABL*-targeted tumours. This reduction in the malignant capacity of the trapped cells corresponded to a lower frequency of Ki-67+ cells within the tumour compared with controls (Figure 6C).

## 3. Discussion

The field of gene therapy for monogenic haematological disorders has advanced significantly in the last two decades, from being a promising strategy to becoming a therapeutic reality. The characteristics of HSC, which can repopulate a patient’s bone marrow and give rise to all haematopoietic lineages, mean that most of these haematological disorders can ultimately be treated by allogenic haematopoietic stem cell transplantation (allo-HSCT) [22]. This standard treatment is the only conventional alternative with the potential to definitively cure these haematological diseases and is, in several cases, the last-resort salvage option [23,24]. However, the lack of suitable compatible donors and associated immunological complications, such as graft-versus-host disease, are serious clinical barriers that limit the success of this treatment and prevent its application in a wide range of haematological diseases [25].

In this context, autologous HSC-based gene therapy benefits from all the experience gained in bone marrow manipulation, autologous and allogenic HSC transplantation and HSCs extraction through CD34+ cell selection, while avoiding all the immunological risks associated with the allo-HSCT process [22,26]. Furthermore, the rapid advances in gene-editing techniques using the CRISPR/Cas9 system and the development of safer therapeutic viral vectors (LVs) that make ex vivo correction of HSCs feasible, have prompted a large number of HSC-based gene therapy clinical trials [27,28,29,30]. Autologous HSC-based gene therapy has demonstrated its curative potential, especially in those pathologies in which gene correction confers a proliferative advantage to those edited cells and, along with the attainment of certain levels of chimerism of genetically modified cells in the graft, is enough to produce full therapeutic benefits [31]. Nevertheless, there are other scenarios, such as the elimination of oncogenes in cancer-therapy, in which achieving certain levels of corrected-cell chimerism is not sufficient.

CML is an excellent example of a haematological malignancy in which all pathological aspects can be attributed to a single oncogene: *BCR/ABL* [10,32]. Our previous study [19] and subsequent others [15,16], have shown that anti-*BCR/ABL* gene therapy could have enormous therapeutic potential, although guaranteeing the absence of residual unedited cells is imperative if this potential is to be realised [18]. A possible solution to this difficulty is to employ a strategy that allows in vitro editing and selection of the HSCs. This approach would overcome the well-established limitations, such as low editing efficiency, and would offer new possibilities, such as in vitro expansion of the virtually pure population of edited cells before their reinfusion into the patient. In this work, we show for the first time an anti-*BCR/ABL* CRISPR-Trap approach that allows the simultaneous genetic correction and expression of a selectable cell marker.

The CRISPR-Trap strategy unites CRISPR/Cas9 technology, which targets the specific *BCR/ABL* fusion sequence, with a non-viral gene trap donor inserted via HDR. The gene trap donor contains a splicing acceptor sequence, which ensures disruption of the *BCR/ABL* reading frame, followed by the *Venus* fluorescent reporter gene sequence. Our preliminary results, using a promoterless SA-T2A-Venus cassette in which *Venus* expression is under the control of *BCR* promoter, showed that the system was able to specifically integrate the cassette, reducing the *BCR/ABL* expression and its oncogenic effect. However, using this strategy, the expression level of *BCR* is not enough to generate detectable fluorescence (Supplementary Materials). Currently, there are new modifications in the gene-trap donor sequence to improve the sensibility of the reporter system, such as enhancers, positive/negative selection systems or tandem repeats of the reporter gene [33,34,35]. However, in all these cases, the increase in the reporter signal was weak and involves a process of selection/expansion of the cells. In the HSC-based therapy, where the expansion of the selected cells is not possible and the use of negative selection genes is not suitable, achieving a robust fluorescent monitoring system becomes imperative.

In this sense, others studies have shown the need to include an exogenous promoter in gene therapy donors to ensure high locus-independent expression of the selection gene [31]. Accordingly, we modify our interfering cassette to led *Venus* expression under the control of the CMV promoter. However, the use of a promoter-containing donor implies that off-target integrations of the cassette, detected in our control conditions, led to an observable expression of the reporter gene [36] obtaining similar fluorescence percentages among all experimental conditions. Interestingly, this result contrasts with that obtained in the *Venus* qPCR quantification, where the sensitivity of the technique revealed large and significant differences in reporter expression levels. There was a more than 13-fold higher level for the donor + CRISPR/Cas9 condition, suggesting an increase in the HDR-mediated integration efficiency of the cassette when CRISPR/Cas9 drives the system.

Our analyses conducted in the Venus fluorescent cell fractions corroborated the adequate selection of the *BCR/ABL*-trapped cells for the donor + CRISPR/Cas9 condition, as well as the unspecific origin (random-integrated and non-integrated donor) of Venus expression under control conditions. Separation of the Venus fluorescent cell fraction from the control conditions did not result in an enrichment of the cell population carrying the trapped *BCR/Venus* allele, nor consequently in a reduction in the frequency of the native *BCR/ABL* allele, thereby revealing locus-independent reporter expression. Moreover it is not possible to distinguish between Venus expression from unspecific integrated donor and from non-integrated free donor, since the K562 cell line depends on *BCR/ABL* to grow in culture and, therefore, it is not possible to carry out the several division rounds needed to get rid of the free donor [37,38]. Conversely, in the CRISPR-mediated group, the selection of Venus fluorescent cells led to an enrichment of the *BCR/ABL*-trapped cell fraction in which the CMV-Venus expression cassette was successfully inserted into the *BCR/ABL* locus. These results are consistent with those of previous reports in which the “on target” HDR-mediated integration of the cassette increased by over 1000-fold when using engineering nucleases capable of generating a double-stranded cut in DNA [39]. This site-specific integration caused the expression of the *BCR/ABL* oncogene, which was already reduced in the set of electroporated cells, to decrease to substantially lower levels in the Venus fluorescent cell fraction.

Finally, we demonstrated the therapeutic benefit of a *BCR/ABL* CRISPR-Trap strategy in vitro and in vivo. The effects of the constitutive *BCR/ABL* activity are pleiotropic and promote leukaemogenesis by acquisition of tumour abilities. These abilities include the increase in cell survival [40,41], apoptosis inhibition [42,43] and genomic instability that downregulates the DNA-repair mechanism [44,45]. In the K562 CML-derived cell line, the CRISPR-Trap system disrupted the *BCR/ABL* sequence, entirely preventing any oncogenic effect on in vitro survival and proliferation in selected Venus fluorescent cells in the CRISPR/Cas9-mediated group. Subcutaneous injection of these *BCR/ABL*-trapped cells into immunodeficient mice led to a strong inhibition of tumour growth, resulting in a 90% reduction in tumour burden relative to control tumours (Cas9 + donor and parental), 23 days post-injection. Earlier results from our group [19] and others [15] were obtained using a strategy based on the induction of aleatory mutations in the reading frame of the oncogene to prevent its expression. In any case, the possibility of inducing mutations without frame-shift consequences, together with the inability to select the edited cells, led to higher-than-desired levels of oncogene expression. By contrast, the CRISPR-Trap system showed, after selecting the edited cells, a more than 80% reduction in *BCR/ABL* expression levels, which represents a substantial improvement in the efficiency of genome editing in those cases where it is possible to select the cells, such as in HSC-based therapies, before transplant.

These results demonstrate that the CRISPR-Trap system achieves adequate selection of *BCR/ABL* null cells, limits the residual unedited events with oncogenic potential and bestows a long-term therapeutic effect in a CML mouse model. Further studies are needed to improve the efficiency of the delivery system, editing, and selection process, but our results represent an advance in gene therapy of haematological genetic diseases and offer a new approach to selecting the correctly edited cells before carrying out an autologous transplant. The CRISPR-Trap design combines established gene trapping strategies, which simultaneously disrupt and report by expression of a selectable marker gene, with the mechanisms of homologous recombination (HR), which direct the system in a site-specific way via designable homology arms. Our study demonstrates the CRISPR-Trap system to be a promising and versatile strategy for gene deletion monitoring that is applicable to any genomic sequence.

## 4. Materials and Methods

### 4.1. Cell Lines and Culture Conditions

The human CML-derived K562 cell line was purchased from the DMSZ collection (Leibniz-Institut DSMZ-Deutsche Sammlung von Mikroorganismen und Zellkulturen GmbH, Germany). K562 cells were cultured in RPMI 1640 medium (Gibco, Thermofisher Scientific, Pleasanton, CA, USA) supplemented with 10% FBS, and 1% penicillin/streptomycin (Thermo Fisher Scientific, Pleasanton, CA, USA) and were incubated at 37 °C in a 5% CO_2_ atmosphere.

### 4.2. Cloning of Targeting Vectors and HDR DNA Donor Obtention

The target vectors pTC201B HA BCR/ABL SA-T2A-CMV-Venus and pTC201B HA BCR/ABL SA-T2A-Venus were generated by modifying the pTC201B vector, kindly provided by Tian Chi [46] (Addgene plasmid # 52193; http://n2t.net/addgene:52193 (accessed on 4 June 2022); RRID:Addgene_52193), containing the Neo-IRES-GFP cassette, preceded by a splicing acceptor sequence (SA). The 5′ and 3′ homology arms of the *BCR/ABL* target site were obtained by PCRs from K562 genomic DNA and subsequently inserted flanking the cassette of the targeting vector using the ClaI and XhoI sites, respectively. The original Neo-IRES-GFP reporter cassette was removed using the NotI and SalI restriction sites and replaced by the CMV-Venus or T2A-Venus constructs obtained by PCR from plasmid pShuttle, kindly provided by Zheng-Xu Wang (Addgene plasmid # 62621; http://n2t.net/addgene:62621 (accessed on 4 June 2022); RRID:Addgene_62621) (Figure 1A; Supplementary Materials Figure S1A).

The double-strand donor DNA molecule (DNA donor), containing the interference cassette (SA-CMV-Venus or T2A-Venus) flanked by the *BCR/ABL* homology arms of 491 and 327 bp, respectively (Figure 1A), was obtained by PCR from the targeting vector using Donor F and Donor R oligonucleotides (Table 1). PCR product was purified using the NZYGelpure kit (NZYTech), digested with DpnI, phosphorylated with T4 polynucleotide kinase, re-purified and eluted in 40 μL of ddH_2_O to a final concentration of 200 ng/μL.

### 4.3. CRISPR/Cas9 System Design

In most CML patients, the BCR/ABL fusion region comprises the major cluster region of *BCR* (M-bcr), downstream of exons 13 or 14, and the region upstream of the second exon (a2) of *ABL* [47,48]. However, the specific genomic breakpoints of both genes involve regions of 3 kb for *BCR* and 200 kb for *ABL*, making each patient’s fusion sequence almost unique.

To specifically target *BCR/ABL*, we sequenced the genomic K562 fusion region and designed a specific sgRNA targeting the *BCR/ABL* junction sequence, using the BreakingCas web-tool (http://bioinfogp.cnb.csic.es/tools/breakingcas/ (accessed on 4 June 2022)) (Figure 1B). The editing efficiency of the sgRNA was assessed in vitro by Sanger sequencing of the *BCR/ABL* genomic region from electroporated cells and analysed by Tracking of Indels by Decomposition (TIDE) software (https://tide-calculator.nki.nl (accessed on 4 June 2022); Netherlands Cancer Institute, Amsterdam, The Netherlands) (Figure 1C).

### 4.4. CRISPR/Cas9 Ribonucleocomplex Assembly, DNA Donor Delivery and Electroporation

The BCR/ABL-sgRNA was prepared by equimolar mixing of the crRNA containing the target sequence (Integrated DNA Technologies, Leuven, Belgium) with the tracrRNA (Integrated DNA Technology, Belgium) to a final concentration of 44 µM. Duplex annealing was carried out by heating at 95 °C for 5 min, followed by a ramp-down of temperature to 25 °C. The ribonucleoprotein complex was obtained by incubating 22 pmol of the previous duplex with 18 pmol of Cas9 enzyme. For each electroporation, we added 1 µL of the ribonucleoprotein, 1 µL of donor DNA (200 ng), 2 μL of 10.8 µM of Electroporation Enhancer (Integrated DNA Technology, Belgium), and 6 μL of cell suspension of 1 × 10^7^ cells/mL.

Cells were electroporated using a Neon Transfection System Kit (Invitrogen, Thermo Fisher Scientific, CA, USA) following the manufacturer’s instructions and using the electroporation parameters of 1450 v, 10 ms and 3 pulses.

### 4.5. DNA/RNA Isolation, Retrotranscription and PCR-Based Detection of HDR Events

Genomic DNA and total RNA from K562 cells were extracted using the AllPrep DNA/RNA Kit (Qiagen) following the manufacturer’s protocol. 100 ng of total RNA was used in an in vitro retrotranscription using SuperScript III First-Strand Synthesis Super Mix kit (Thermo Fisher Scientific, CA, USA). DNA and cDNA were amplified in several PCRs, using different pairs of primers for specific 5′ (Out 5′F and Venus R) and 3′ (Venus F and Out 3′R) integration junction, in order to detect targeted integration of the DNA donor or unspecific cDNA expression (In 5′F and Venus R; Table 1). PCR and RT-PCR were performed using the Phusion Taq polymerase (Thermo Fisher). All PCR products were cleaned up using an NZYGelpure kit (NZYTech, Lisbon, Portugal) and directly sequenced by the Sanger method using forward and reverse PCR primers.

### 4.6. Cell Viability and Cell Proliferation Assay

Cell viability was determined by annexin V-Dy634 (apoptosis-detection kit ANXVVKDY, Immunostep, Spain). A total of 5 × 10^4^ pool or Venus+ sorted cells, according to the experimental design, were seeded in 48-well plates after electroporation. At each time point, cells were harvested, washed twice in PBS and labelled with annexin V (AV) staining, which enabled living cells to be identified.

For cell proliferation assays, 1.5 × 10^4^ cells from the electroporated cell pool or from the Venus+ cell fraction were seeded 24 h after electroporation. Cells were harvested at 48, 72 and 96 h, and the number of live cells was counted by flow cytometry. These experiments were performed using FACScalibur (BD Biosciences, San Jose, CA, USA) and the data were analysed using FlowJo software (vX.0.7. TreeStar, Woodburn, OR, USA).

### 4.7. Flow Cytometry and Cell Sorting

K562 cells were analysed by fluorescence-activated cell sorting (FACS) using FACSaria (BD Biosciences, CA, USA) 24 h after electroporation with the CRSPR/Cas9 reagents and the donor DNA. Cell sorting was used to select the gene-targeted cells by separation of the Venus+ cell population. Results were analyzed using FlowJo software.

### 4.8. qPCR

Expression levels of *BCR/ABL*, *BCR/Venus* and *Venus* were measured by qPCR using the cDNA from gene-targeted cells and SYBR Green Master Mix (Applied Biosystems). Expression levels were normalized relative to the GAPDH housekeeping gene. qPCR was performed and data were analysed using the StepOnePlus™ system (Applied Biosystems) (Table 1).

### 4.9. Mouse Xenograft Tumorigenesis

Ten 4–5-week-old female NOD-scid IL2Rgammanull mice (Strain #005557) (Charles River, Barcelona, Spain) were used (five mice per group). Tumour xenografts were induced by subcutaneous injection of cell suspensions containing 4 × 10^5^ cells in 0.1 mL of RPMI medium and 0.1 mL of Matrigel^®^ (Corning^®^ Matrigel^®^ Basement Membrane Matrix) into the mouse flank. This study followed the Spanish and European Union guidelines for animal experimentation (RD 1201/05, RD 53/2013 and 86/609/CEE, respectively). The study received prior approval from the Bioethics Committee of our institution.

Before injection, 4 × 10^5^ Venus+ cells were sorted by FACSaria (BD systems) from Cas9 + donor or CRISPR/Cas9 + donor electroporated cells. The same number of live cells were sorted in parental K562 cells and used as a control. Cells were counted using a Neubauer chamber (VWR) and cell viability monitored by trypan blue staining (Sigma).

In the first group of mice, K562 parental cells were injected into the left flank and Cas9 + donor sorted cells into the right flank; in the second group, K562 parental cells were injected into the left flank and CRISPR/Cas9 + donor sorted cells into the right flank. Tumours were measured on days 9, 12, 15, 19, 22 and 23, and their volume calculated as described elsewhere [49] by the formula a2bπ/6 (where a and b are, respectively, the smallest and the largest diameters). Mice were sacrificed by anaesthesia overdose 23 days after cell injection, whereupon the tumours were collected and weighed.

### 4.10. Immunohistochemical Studies

Tumour tissues were fixed with 10% formaldehyde in PBS overnight at RT and paraffin-embedded. Tissue sections of 2 µm were deparaffinized, rehydrated, blocked with 3% hydrogen peroxide (Merck), subjected to heat-induced antigen retrieval, and incubated with a 1:50 dilution of rabbit anti-Ki-67 (Roche) and an Omnimap Rabbit secondary antibody (Roche). Immunostained preparations were revealed with DAB and counterstained with haematoxylin.

### 4.11. Statistical Analysis

Statistical analyses were performed using GraphPad Prism 6 Software (GraphPad Software). Group differences between annexin V labelling levels were assessed with Mann-Whitney U tests. Differences in *BCR/ABL* and *BCR/Venus* expression levels among different groups were estimated by Student’s *t*-tests or two-way ANOVAs with Tukey multiple post hoc comparisons. Statistical significance of different levels was concluded for values of *p* < 0.05 (*), *p* < 0.01 (**) and *p* < 0.001 (***).

## Figures and Tables

**Table 1:** Oligonucleotides used.

Name	Sequence
Donor F	ACCCACATCCCACATCACCC
Donor R	CATGGTCTCCACTATCAAGGG
Out 5′ F	ATCAAGGATCTCCGGGCAGC
Out 3′ R	CCAAGGCAAATCTGGGAGTTG
In 5′ F	TCCACTCAGCCACTGGATTTAAGCA
Venus F	TGGTCCTGCTGGAGTTCGTG
Venus R	GGACACGCTGAACTTGTGGC
BCR qPCR F	AGTTACACGTTCCTGATCTCC
ABL qPCR R	TTGGGCTTCACACCATTCCCC
CMV qPCR R	GCGGGCCATTTACCGTAAG
Venus qPCR R	GCGGGCCATTTACCGTAAG
Gapdh qPCR F	TGCACCACCAACTGCTTAGC
Gapdh qPCR R	CACCACCTTCTTGATGTCATCA

## Supplementary Materials

The following supporting information can be downloaded at: https://www.mdpi.com/article/10.3390/ijms23126386/s1.
