# Supplementary material for: CRISPR/Cas9-Directed Gene Trap Constitutes a Selection System for Corrected BCR/ABL Leukemic Cells in CML
Source: Int J Mol Sci. 2022 Jun 7;23(12):6386. doi: 10.3390/ijms23126386 (PMC9224210; doi:10.3390/ijms23126386)
Supplement: Supplementary file 1 [file ijms-23-06386-s001.zip › ijms-1729808-supplementary.pdf]

Supplementary material.

CRISPR/Cas9-directed gene trap constitutes a selection system for corrected *BCR/ABL* leukemic cells in CML

Elena Vuelta [1,2,4,6], José Luis Ordoñez [3,6], David J Sanz [2], Sandra Ballesteros [1,2], Jesús María Hernández-Rivas [1,2,5,6], Lucia Méndez-Sánchez [4], Manuel Sánchez-Martín [1,4,6] * and Ignacio García-Tuñón [1,2,6] *.

Results

The promoter-less CRISPR-Trap system efficiently the specific integration of the gene trap donor in BCR/ABL but is not a robust reporter system to select corrected cells.

As a first approach, we generate a dsDNA donor by PCR amplification containing a SA-T2A-Venus interfering cassette (Figure S1A). The K562 cell line was divided into three experimental groups according to the conditions for their subsequent electroporation: a) with the donor dsDNA (donor), b) with the donor DNA and Cas9 nuclease without sgRNA (Cas9 + donor) and c) with the donor DNA, Cas9 nuclease and the specific *BCR/ABL* sgRNA (CRISPR/Cas9 + donor). Twenty-four hours after electroporation, no fluorescent cells (*Venus* + cells) were observed in any of the three groups (Figure S1B).

The result of the site-unspecific PCR (In 5´F/Venus R, Table 1) which amplify a region contained entirely in the donor HDR dsDNA, showed a band of the expected size in the Cas9 + donor and CRISPR/Cas9 + donor groups, but not in the donor group (Figure S1C), implying that all donor HDR dsDNA not integrated in the genome was fully degraded. In contrast, the site-specific PCR (Out 5´F/Venus R, Table 1) corroborated the correct insertion of the donor HDR dsDNA at the *BCR/ABL* target sequence only in the CRISPR/Cas9 + donor group, with no site-specific integration detected in any of the controls (Figure S1C). These results were corroborated by Sanger sequencing of the PCR products, supporting the HDR-mediated insertion of the donor dsDNA into the CRISPR/Cas9 + donor group.

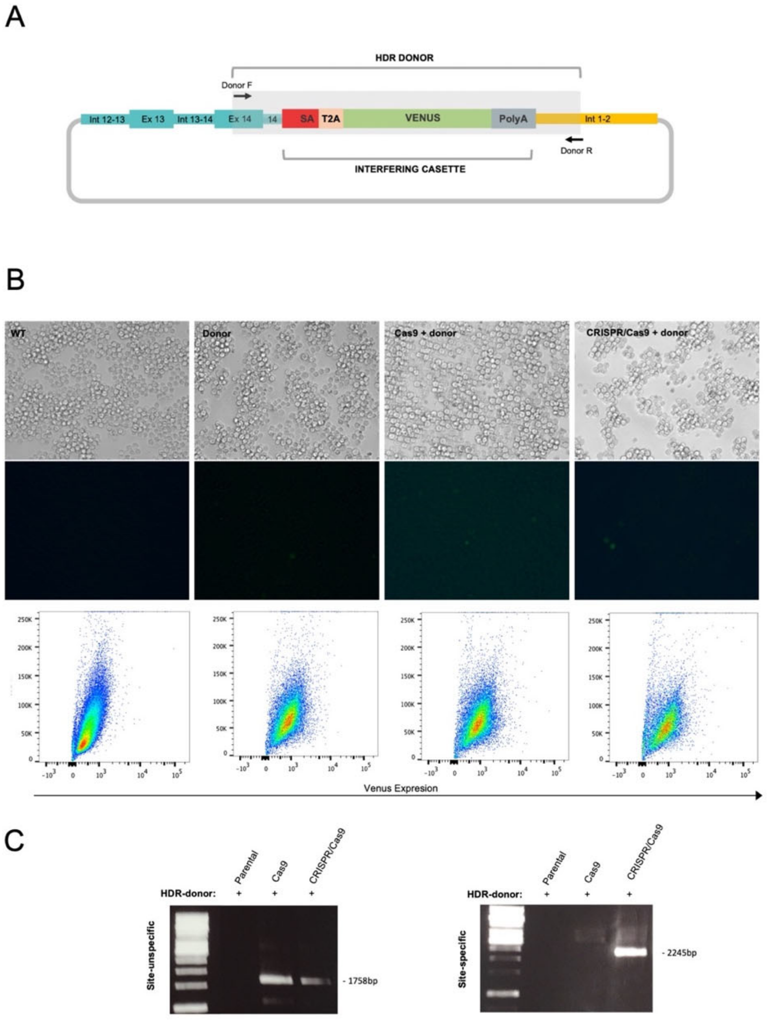

Supplemental Figure S1. (A) Schematic representation of CRISPR-Trap system plasmid and the target region of *BCR/ABL* fusion gene. Plasmid harboring the interfering cassette flanked by homology arms, containing a splicing acceptor sequence (SA), T2A sequence and the Venus fluorescent protein coding sequence. CRISPR-Trap system electroporation in K562 cells. **(B)** *Venus* expression K562 electroporated with HDR donor (used as control) and CRISPR-Trap system (CRISPR/Cas9-HDR donor) and Venus-positive cell quantification by flow cytometry of each condition. **(C)** Molecular characterization of CRISPR-Trap system. 5´junction PCR site-unspecific and specific. Site-unspecific PCR amplified a 1758 bp (oligos: In5´F/VenusR) amplification and in cells electroporated with CRISPR-Trap system and controls (Parental, Parental + Donor, Cas9 + donor). Site-specific PCR amplification in cells electroporated with CRISPR-Trap system (oligos: Out5´F/VenusR; 2245 bp).

To explore the proper functionality of the CRISPR-Trap system, we studied all the possible mRNAs generated from the trapped allele. We designed a pair of oligonucleotides to amplify a region of the donor DNA (BCR qPCR F/Venus R; Table 1). This in-in RT-PCR showed a 557-bp band, corresponding to the size of the predicted *BCR/Venus* mRNA specifically inserted into the *BCR/ABL* locus, only with the CRISPR/Cas9 + donor condition (Figure S2A). Interestingly, Sanger sequencing of the PCR products confirmed the existence of a processed BCR-Venus mRNA (data not shown).

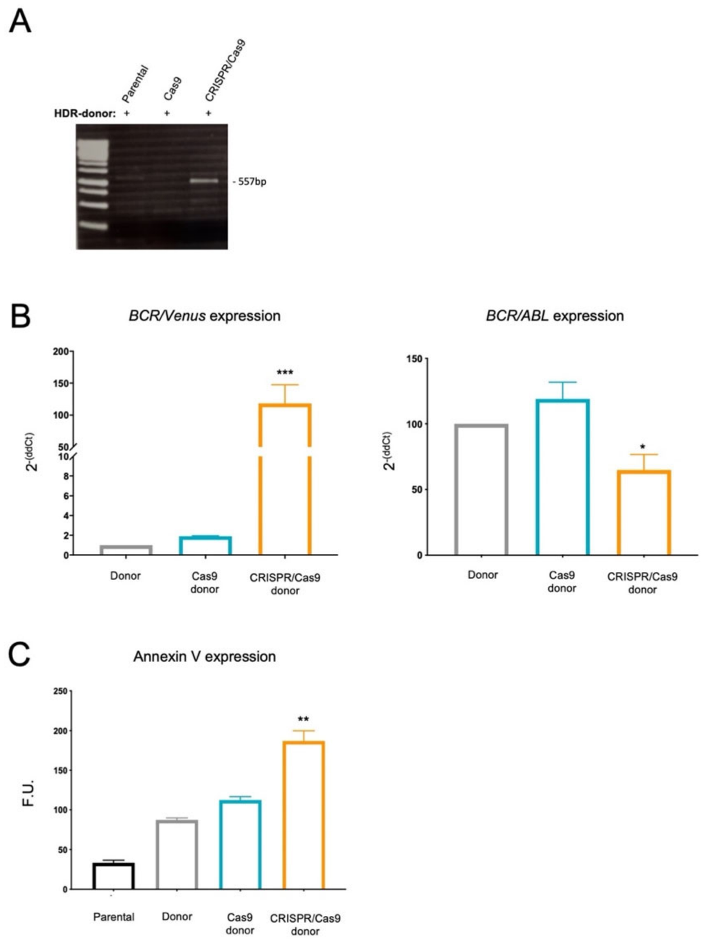

Supplemental Figure S2. (A) Expression analysis of target locus. RT-PCR of *BCR/Venus* fusion RNA (557 bp site-specific, oligos: BCRqPCRF/VenusR), in cells electroporated with the CRISPR-Trap system (CRISPR-Cas9 + HDR donor) and controls (Parental, Parental + donor, Cas9 + donor). **(B)** qPCR of *BCR/Venus* and **(B)** BCR/ABL in K562 cells electroporated with the CRISPR-Trap system (CRISPR/Cas9 + donor) and controls (donor and Cas9 + Donor). **(C)** Functional analysis of CRISPR-Trapped *BCR/ABL*. Annexin V expression analysis by flow cytometry of K562 cells 48 hours after electroporation with the CRISPR-Trap system (CRISPR/Cas9 + Donor) and controls (Parental, Parental + Donor, Cas9 + donor) (mean ± SEM; **, $p<0.01$; ***, $p<0.001$).

Once demonstrated the expression of the interfering cassette, we proceeded to quantify the expression levels of *BCR/ABL* trapped locus versus native *BCR/ABL* allele in electroporated cells using a common forward oligonucleotide in the *BCR* sequence (BCR qPCR F) and two reverse oligonucleotides hybridizing in the ABL (ABL qPCR R) or CMV differential sequence (CMV qPCR R), respectively (Table 1).

BCR/Venus qPCR confirmed the significant expression of the *BCR-Venus* allele solely in the CRISPR/Cas9 + donor group, in which it attained expression levels 118-fold greater than in the controls groups (Figure S2B), while the BCR/ABL qPCR showed significantly lower oncogene mRNA levels in cells electroporated with the CRISPR-Trap system relative to control groups, (Figure S2B).

To evaluate the biological effect of the *BCR/ABL* CRISRP-Trap strategy, we measured the apoptotic levels and proliferative capacity of each group of electroporated cells. Forty-eight hours

after electroporation, Annexin-V staining showed no significant differences between parental K562 cells (33.3 uf) and control groups (87.3 and 112.4 uf with the donor and Cas9 + donor conditions, respectively). In contrast, cells electroporated with the entire CRISPR-Trap system showed significantly higher levels of Annexin-V (186.8 uf), with respect to all control groups (Figure S2C).

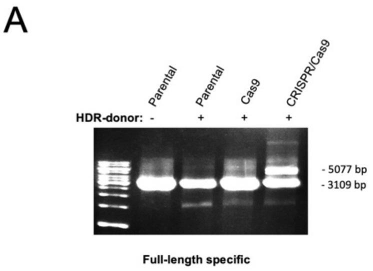

Supplemental Figure S3. (A) Molecular characterization of CRISPR-Trap system. Full-length PCR site-specific (oligos: Out 5´F/Out 3´R). PCR amplified a 3109 bp band in all conditions (Parental, Parental + Donor, Cas9 + donor) corresponding to wild type *BCR/ABL* allele. In cells electroporated with CRISPR-Trap system we observed and extra band of 5077 bp corresponding to trapped allele.
